# Digital Monitoring of Pre-Exposure Prophylaxis Users Through Electronic Patient-Reported Outcome Measures and Electronic Patient-Reported Experience Measures: Multicenter Prospective Study on Feasibility, Safety, and Predictive Modeling of Digital Engagement

**DOI:** 10.2196/87592

**Published:** 2026-04-13

**Authors:** Gabriel Mercadal-Orfila, Joaquín Ignacio Serrano, Tilman Mijares, Lluís Vidal, Adrian Curran, Salvador Herrera-Pérez

**Affiliations:** 1 Hospital General Mateu Orfila, Mahon, Balearic Islands, Spain; 2 Hospital Son Llatzer, Mahon, Balearic Islands, Spain; 3 Valencian International University, C. del Pintor Sorolla, 21, Ciutat Vella, Valencia, 4500, Spain, 34 630-656-656

**Keywords:** pre-exposure prophylaxis, digital follow-up, health-related quality of life, patient engagement, electronic patient-reported experience measure, electronic patient-reported outcome measure

## Abstract

**Background:**

Digital health tools integrating electronic patient-reported outcome and experience measures (ePROMs/ePREMs) enable longitudinal monitoring of health-related quality of life (HRQoL), psychological well-being, and treatment satisfaction in pre-exposure prophylaxis (PrEP) users. However, determinants of sustained engagement with digital follow-up platforms remain insufficiently characterized.

**Objective:**

To describe the feasibility of the Naveta-Phemium digital platform for longitudinal monitoring of safety, HRQoL, and treatment satisfaction among PrEP users, and to develop and internally validate a machine learning framework to characterize and predict engagement with digital follow-up.

**Methods:**

A prospective observational study was conducted using the Naveta digital follow-up platform. HIV-negative adults at high risk of HIV infection received tenofovir disoproxil fumarate (245 mg) plus emtricitabine (200 mg). Clinical safety, HRQoL, and satisfaction were assessed using laboratory parameters and validated ePROMs/ePREMs (Hospital Anxiety and Depression Scale, Patient-Reported Outcome Measurement Information System Profile-29, Treatment Satisfaction Questionnaire for Medication, and Person-Centered Coordinated Care Experience Questionnaire). Engagement was defined at the questionnaire level and analyzed using the ALGOPROMIA-Classification framework with repeated stratified cross-validation. Model explainability was assessed using permutation-based Shapley Additive Explanations.

**Results:**

A total of 81 participants contributed repeated questionnaire-level observations (mean PrEP duration 689 d). PrEP was well tolerated, with no moderate or severe adverse events; mild transient symptoms were mainly gastrointestinal (31/45, 68.9%) and neurological (26/45, 57.8%). Renal function remained stable (creatinine: 0.86 [SD 0.13] mg/dL; estimated glomerular filtration rate: *P*=.498). Psychological well-being and HRQoL remained stable (Hospital Anxiety and Depression Scale<7; Patient-Reported Outcome Measurement Information System Profile-29 near population norms). Treatment satisfaction was consistently high (Treatment Satisfaction Questionnaire for Medication≈85‐87), and satisfaction with the NAVETA telemedicine model remained stable (8/10). Engagement showed clear sociodemographic and behavioral gradients. Ensemble-based machine learning models achieved good discrimination in predicting engagement (area under the curve≈0.82) across ≈12,300 questionnaire-level observations. Random forest was retained for robustness and consistency. Shapley Additive Explanations analysis highlighted lifestyle-related variables as the most influential predictors, with heterogeneous individual-level effects.

**Conclusions:**

Naveta enabled feasible telemedicine-based PrEP follow-up with preserved HRQoL, high satisfaction, and stable safety. Combining longitudinal ePROMs/ePREMs with explainable machine learning allowed detailed characterization of digital engagement, supporting digitally supported PrEP care optimization and informing future comparative and cost-effectiveness studies.

## Introduction

HIV infection continues to be a stigmatized condition in many social contexts. In Spain, pre-exposure prophylaxis (PrEP), based on outpatient hospital pharmacy medicines, has proven to be an effective and safe strategy for HIV prevention among populations at high risk of infection [1]. Following the recommendation of the European Medicines Agency, PrEP with emtricitabine/tenofovir disoproxil was authorized in Spain in August 2016 for HIV-negative individuals aged 16 years or older at high risk of acquisition [2]. In routine clinical practice, this regimen has been consistently reported as safe and well-tolerated [3-5]. Nevertheless, PrEP does not replace other preventive measures for HIV or other sexually transmitted infections [6,7] and must be prescribed within specialized units as part of a comprehensive HIV prevention strategy.

Beyond clinical effectiveness and safety, contemporary health care increasingly emphasizes a more integrated perspective that incorporates health-related quality of life (HRQoL) across physical, emotional, and social dimensions. For individuals using PrEP, this approach is particularly relevant, as persistent stigma surrounding HIV prevention may influence psychological well-being, engagement with health care services, and participation in digital follow-up programs [8]. Previous studies have shown that higher physical, psychological, and environmental HRQoL scores are associated with a greater likelihood of PrEP use [9]. Accordingly, in addition to clinical outcomes, it is important to assess treatment-related satisfaction and HRQoL, as these factors may influence adherence to preventive therapy [10]. Although a positive impact of PrEP on HRQoL has been described [1], further studies using validated assessment frameworks are needed to generate reliable evidence and to support strategies aimed at sustaining adherence to PrEP over time [11-14].

Digital health technologies, particularly telemedicine [15,16], combined with electronic patient-reported outcome measures (ePROMs) and electronic patient-reported experience measures (ePREMs) [17], represent a promising approach to address these needs within person-centered health care models [18]. Digital interventions have demonstrated substantial potential in HIV prevention and care [19], including among individuals with mental health conditions or substance use disorders [20,21], where ePROMs may facilitate early identification of vulnerability [22]. However, adherence to digital prevention and follow-up programs remains highly variable [23-26]. Engagement in such programs is shaped not only by clinical factors but also by psychosocial, demographic, and behavioral determinants, including age, gender, educational level, and health-related behaviors, whose interrelationships are often complex and nonlinear [27-29].

Sustained engagement is critical for the validity of longitudinal digital monitoring and for translating real-world patient-reported data into meaningful clinical insights [30-32]. High engagement enhances the reliability of ePROMs and ePREMs and supports value-based health care strategies by enabling more informed, patient-centered decision-making [33,34]. However, classical statistical approaches may be insufficient to capture the multidimensional and nonlinear patterns underlying digital engagement, particularly when multiple patient-reported measures are considered simultaneously. In this context, machine learning methods provide a robust alternative, as they are well-suited to modeling complex interactions, handling high-dimensional data, and improving discrimination beyond traditional regression techniques.

Within this framework, the Naveta value-based telemedicine initiative has recently been developed as a digital platform for collecting ePROMs and ePREMs [35]. The Naveta-Phemium platform has demonstrated efficiency in chronic disease populations and may be equally applicable to individuals receiving PrEP, who represent preventive users rather than patients. In parallel, the ALGOPROMIA project is an ongoing initiative focused on predicting patient engagement—operationalized as response rates to ePROMs and ePREMs—using machine learning models applied to data generated within the Naveta digital HRQoL follow-up program [36].

On the basis of these considerations, the objectives of this study were twofold. The primary objective was to describe the usefulness of the Naveta-Phemium platform for longitudinal monitoring of effectiveness, safety, HRQoL, and treatment satisfaction among HIV-negative adults receiving PrEP over a 24-month follow-up period. The secondary objective, using the ALGOPROMIA framework, was to develop and internally validate a machine learning–based participant classification system to characterize and predict engagement with the digital follow-up tool among PrEP users.

## Methods

### Study Design and Participants

This was an observational multicenter study conducted in two public hospitals in the Balearic Islands (Spain). All HIV-negative adults at high risk of HIV infection who were starting or continuing PrEP between November 2022 and March 2025 were considered eligible. Participants were required to be registered in the Naveta-Phemium PrEP HIV program, as previously described by Mercadal-Orfila et al [35], and to provide written informed consent. Participants who provided informed consent (refer to the Ethical Considerations subsection for details) received a link to access the electronic questionnaires, which they could complete on their own device (PC, tablet, or mobile phone). Participants for whom data could not be obtained because of incomplete medical records were excluded from the study.

Participants were recruited consecutively during routine clinical care through the Hospital Pharmacy Service within specialized PrEP units at the participating public hospitals. Eligible individuals were informed about the study by hospital pharmacists as part of standard care procedures and were invited to participate without additional selection criteria. Participation was voluntary, no incentives were offered, and no replacement or targeted sampling strategies were applied.

### Ethical Considerations

This study was approved by the Ethics Committee for Research with Medicines of the Balearic Islands (Comitè d’Ètica de la Investigació amb Medicaments de les Illes Balears, CEIm), under protocol code IB 5117/23 EOm. The committee issued a favorable opinion for this prospective observational study with medicinal products, including approval of the recruitment period extension, in accordance with Spanish regulations governing observational studies with medicines (Royal Decree 957/2020). The study was conducted in compliance with the principles of the Declaration of Helsinki and Good Clinical Practice guidelines.

All participants provided informed consent prior to inclusion in the study through a secure electronic process implemented via the NAVETA platform. Participants accessed the study information and consent materials through a web-based link and electronically provided informed consent. The informed consent documents, participant information materials, and electronic consent procedures were reviewed and approved by the Ethics Committee for Research with Medicines of the Balearic Islands (CEIm), in line with the observational nature of the study.

Participant data were processed in compliance with applicable data protection regulations. All data used for analysis were deidentified prior to processing, and access to identifiable information was restricted to authorized clinical personnel only. Data were stored on secure servers with appropriate technical and organizational safeguards to ensure confidentiality and prevent unauthorized access.

No financial compensation or material incentives were provided to participants for their participation in this study.

### Recruitment, Data Collection, and Variables

Patients receiving PrEP were initially assessed and clinically managed by the Infectious Diseases service at public hospitals in the Balearic Islands, Spain, in accordance with established clinical protocols [37]. Once patients met the eligibility criteria and were referred to outpatient hospital pharmacy services for medication dispensing and follow-up, recruitment for the telemonitorization program was conducted exclusively within the hospital pharmacy setting.

During routine outpatient pharmacy consultations, hospital pharmacists informed eligible adult patients—either at PrEP initiation or during routine follow-up—about the objectives of the telemonitorization program and the procedures involved. Patients who expressed interest were provided with a secure link granting access to the NAVETA platform.

Through this platform, participants reviewed the study information, accepted the study conditions, and provided informed consent via an electronic consent process. Following confirmation of consent, participants initiated the telemonitorization process and completed the electronic questionnaires in accordance with the NAVETA_VIH protocol.

Sociodemographic and lifestyle characteristics were collected through an ad hoc questionnaire integrated into the Naveta-Phemium platform. This questionnaire captured information on age, gender, marital status, educational level, employment status, patient association membership, consumption habits, health-related behaviors, drug allergies, and PrEP treatment line. Health-related behaviors included BMI, self-reported adherence to a balanced diet, and level of physical activity, as defined in the Naveta platform [35].

Clinical safety data related to emtricitabine/tenofovir disoproxil were extracted from routine hospital medical records. These included adverse events, treatment discontinuations, serum creatinine levels, and estimated glomerular filtration rate (eGFR, mL/min/1.73 m^2^), calculated using the CKD-EPI equation, as well as hepatitis infections, HIV seroconversions, and sexually transmitted infections. Clinical data were collected at baseline and at 6, 12, 18, and 24 months of follow-up.

### Assessment Instruments and Follow-Up Schedule

Patient-reported outcomes and experiences were assessed longitudinally using validated instruments: the Treatment Satisfaction Questionnaire for Medication (TSQM) [38], the Patient-Reported Outcome Measurement Information System Profile-29 (PROMIS-29) [39], the Hospital Anxiety and Depression Scale (HADS), and the Person-Centered Coordinated Care Experience Questionnaire (P3CEQ) [40].

Data collection followed the predefined assessment schedule of the Naveta follow-up program. HADS and TSQM were administered at baseline and at 6, 12, 18, and 24 months, whereas PROMIS-29 and P3CEQ were completed at baseline, 12, 18, and 24 months.

### Statistical Analysis

All statistical analyses, including descriptive statistics, inferential tests, and machine learning procedures, were conducted in Python (version 3.10) [41]. Baseline demographic and clinical characteristics were summarized using descriptive statistics. Continuous variables are reported as mean (SD), and categorical variables as frequencies and percentages.

Associations between digital engagement and sociodemographic or lifestyle variables were explored by comparing engagement scores (mean, SD) across categories (eg, gender, age range, employment status, BMI, lifestyle habits, and educational level). Correlations among ePROMs were assessed using the Spearman correlation coefficient.

Group comparisons were performed using parametric or nonparametric tests according to distributional assumptions. Normality of continuous variables was assessed using the Shapiro–Wilk test, and homoscedasticity was evaluated using the Levene test. When both assumptions were met (*P*>.05 for Shapiro–Wilk and Levene tests), one-way ANOVA followed by Tukey post hoc test was applied. When these assumptions were not fulfilled, nonparametric alternatives were used: the Kruskal–Wallis test with Dunn post hoc comparisons for variables with more than two categories, and the Mann–Whitney *U* test for dichotomous variables (eg, gender, substance use). For all Kruskal–Wallis post hoc analyses, multiple comparisons were adjusted using the Dunn test with Bonferroni correction.

For longitudinal visualization of engagement rates, observed proportions were accompanied by 95% CIs calculated using the Wilson score method, which provides improved coverage for binomial proportions, particularly when sample sizes vary across time points.

### Longitudinal Analysis and Missing Data Handling

Longitudinal analyses accounted for variation in sample size across follow-up timepoints, reflecting the asynchronous recruitment on 07/01/2026 05:15:00 PM and follow-up inherent to the real-world design. At each time point, the effective sample size corresponded to participants who completed the relevant questionnaires. Missing data were handled using case-wise exclusion, retaining all available observations for each specific analysis. Completion of ePROMs and ePREMs within the Naveta platform is voluntary at each scheduled timepoint, resulting in intermittent and participant-specific missingness patterns. Because this missingness structure does not satisfy the assumptions required for multiple imputation, case-wise exclusion was considered the most conservative and assumption-free approach. To assess whether noncompletion reflected systematic differences between participants, an exploratory comparison of baseline ePROMs scores was performed between individuals with low completion (≤25% of scheduled assessments) and high completion (≥75%). The results of this analysis are reported in the Results section. No outliers were removed or adjusted, and all analyses reflect the natural variability of the clinical population.

For engagement analyses, Shapiro–Wilk tests indicated nonnormal score distributions, and Levene tests revealed heteroscedasticity across comparison groups. Accordingly, nonparametric methods were applied using the Kruskal–Wallis test with Dunn post hoc comparisons. For longitudinal and subgroup-level comparisons, the primary objective was descriptive characterization rather than formal hypothesis testing. Results are therefore reported using effect direction, distributional patterns, and associated *P* values, without emphasis on full test statistics (eg, F, H, or W values), which are of limited interpretability in the presence of unbalanced repeated observations, heteroscedasticity, and intermittent missingness. This reporting strategy is consistent with recommendations for observational digital health studies, where statistical inference is used to support descriptive trends rather than to test predefined causal hypotheses.

For qualitative interpretation of scores, established thresholds were applied where available. For HADS, subscale scores were interpreted using standard cut-offs: 0‐7=normal, 8‐10=borderline, and ≥11=clinical case [42,43]. PROMIS-29 T-scores (mean 50, SD 10) were interpreted using provisional thresholds of approximately 55 (mild), 60 (moderate), 65 (moderately severe), and 70 (severe) for relevant domains [44]. For TSQM and P3CEQ, no definitive cut points are available for Spanish populations; therefore, scores were interpreted relative to sample distributions and existing literature.

### Machine Learning Analysis: ALGOPROMIA-Classification

Although clinical outcomes were assessed according to a predefined 24-month follow-up schedule, analyses of digital engagement and model stability were conducted at the questionnaire level and therefore incorporated all available observations generated within the platform, including those occurring beyond the formal clinical follow-up window. Digital engagement was operationalized as a binary outcome, and participants were classified as “good responders” or “poor responders” based on their mean completion rate of scheduled ePROMs and ePREMs across follow-up (threshold ≥0.50). Three machine learning algorithms were evaluated—histogram-based gradient boosting (HGB), random forest (RF), and logistic regression—given their suitability for binary classification and their ability to model both linear and nonlinear relationships [45].

All predictors were transformed using one-hot encoding, and model hyperparameters were selected according to recommendations from prior work [46]. Importantly, the engagement outcome was defined exclusively by response occurrence to scheduled questionnaire dispatches, independently of whether questionnaire content was completed. Thus, missing ePROMs/ePREMs data did not contribute to outcome definition, avoiding circularity between predictor availability and the target variable.

Because of class imbalance, the RandomOverSampler method was applied exclusively within the training folds to prevent information leakage. Model performance was evaluated using repeated stratified 5-fold cross-validation (five repeats), replacing an initial single train–test split. Oversampling was conducted only within training folds, and evaluation was performed on untouched test partitions. Performance metrics are reported as mean and SD of receiver operating characteristic-area under the curve (ROC-AUC), accuracy, precision, recall, and *F*_1_-score across folds. This framework mitigates overfitting in small real-world datasets. The ALGOPROMIA framework has previously been applied in people living with HIV [47], although this does not constitute external validation for the present PrEP cohort.

To characterize score separation between engaged and nonengaged observations, the distribution of predicted probabilities was examined using kernel density estimates. Predicted probabilities from the final model were stratified by observed engagement status, and smoothed density curves were used to visualize overlap and separation between classes across the full range of decision thresholds.

### Model Explainability

After internal validation, a final model was retrained using the fully oversampled dataset to enable interpretability analysis. Feature contributions were examined using SHAP (Shapley Additive Explanations), using a permutation-based estimator compatible with ensemble models [48].

SHAP values quantify each predictor’s contribution to the model output in log-odds space. Mean absolute SHAP values were used to assess overall feature importance independent of direction, whereas mean signed SHAP values indicated the predominant direction of association (positive or negative) across observations. A feature may therefore show a high mean absolute impact with a near-zero mean signed value when its effect varies in direction across individuals. Consistent with current interpretability guidelines, SHAP values were interpreted as indicators of relative importance and directional influence rather than as absolute changes in predicted probability.

Although the cohort comprised 81 individuals, the classifier was trained on the full set of questionnaire-response events. Because engagement was defined at the questionnaire instance level, the effective sample size reflected repeated observations per participant rather than the number of individuals.

## Results

### Study Cohort Characteristics, Clinical and Sociodemographic Profile

Our study included 81 (93.1%) individuals enrolled in the Naveta-Phemium PrEP VIH initiative who agreed to participate. A total of 6 (6.9%) individuals declined participation. The most frequently reported reason was lack of interest in participating (3/6, 50%), followed by unspecified reasons (2/6, 38.2%) and limited access to a mobile phone, email, or internet (1/6, 11.8%).

The majority were male (77/81, 95.1%), with most participants (60/81, 74.1%) aged between 21 and 45 years. Regarding social characteristics, 74.3% (n=55) were single, 46.9% (n=38) held a bachelor’s degree or higher, and 79.0% (n=64) were employed. Among the health-related behaviors, 54.3% (n=44) have a normal body weight, 48.3% (n=29) reported following a regular balanced diet, while 46.7% (n=28) engaged in physical activity regularly, and another 46.7% (n=27) did so occasionally. Regarding the consumption habits, 48.3% (n=29) had never smoked, and the majority reported occasional (47/81, 58.0%) or weekend (20/81, 24.7%) alcohol consumption. Use of other substances was low, with 85.3% (n=64) reporting no consumption (Table 1). Comorbidities were present in 14.3% of PrEP users. The most frequent condition was hypertension (7.6%), followed by other comorbidities (5.9%) and hypercholesterolemia (3.4%).

**Table 1.** Sociodemographic, lifestyle, and health-related characteristics of adult patients receiving HIV pre-exposure prophylaxis (PrEP) enrolled in a prospective observational study conducted at public hospitals in the Balearic Islands (Spain), 2023‐2024 (N=81).

Participant characteristics	Values, n (%)^a^
Sex	
Female	4 (4.9)
Male	77 (95.1)
Age range (years)	
66‐75	2 (2.5)
46‐65	19 (23.5)
21‐45	60 (74.1)
Marital status	
Widowed	1 (1.4)
Divorced	4 (5.4)
Unknown	2 (2.7)
Married/Cohabiting	12 (16.2)
Single	55 (74.3)
Education level	
Primary education	3 (3.7)
Secondary education	18 (22.2)
Vocational training	21 (25.9)
Bachelor’s degree	38 (46.9)
PhD	1 (1.2)
Employment status	
Retired	2 (2.5)
Temporary leave	3 (3.7)
Unemployed	6 (7.4)
Student	6 (7.4)
Employed	64 (79.0)
Not belonging to the patients’ association	81 (100)
Consumption habits	
Alcohol consumption	
Daily	5 (6.2)
Weekends	20 (24.7)
Occasionally	47 (58)
Never	9 (11.1)
Tobacco use	
Yes	17 (28.3)
Ex-Smoker	14 (23.3)
Never	29 (48.3)
Other substances (abuse drugs)	
Yes	11 (14.7)
No	64 (85.3)
Health-related behaviors	
BMI range	
Unknown/Not Classified	1 (1.2)
Obesity	10 (12.3)
Overweight	26 (32.1)
Normal weight	44 (54.3)
Balanced diet	
Never	1 (1.7)
Occasional	30 (50.0)
Regular	29 (48.3)
Physical activity	
Never	4 (6.7)
Occasional	28 (46.7)
Regular	28 (46.7)
Drug allergies	
Yes	6 (7.4)
No	75 (92.6)

a n (%): Absolute and relative frequencies. The total number of responses varies due to nonmandatory fields and missing data.

All participants received emtricitabine/tenofovir 200/245 mg, with a mean treatment duration of 688.9 days (95% CI 602.3‐775.5). During follow-up, 16 out of 81 patients (19.8%) discontinued or interrupted PrEP treatment, a finding partly associated with the use of on-demand rather than continuous regimens. Importantly, no discontinuations were attributed to adverse effects. Regarding emtricitabine/tenofovir effectiveness, no evidence of HIV seroconversions during the period of 24 months was found. On the other hand, some types of sexually transmitted infections were reported in 35.9% of subjects, highlighting *Neisseria gonorrhoeae* (30.4%) and *Chlamydia trachomatis* (13%).

**Figure 1.**
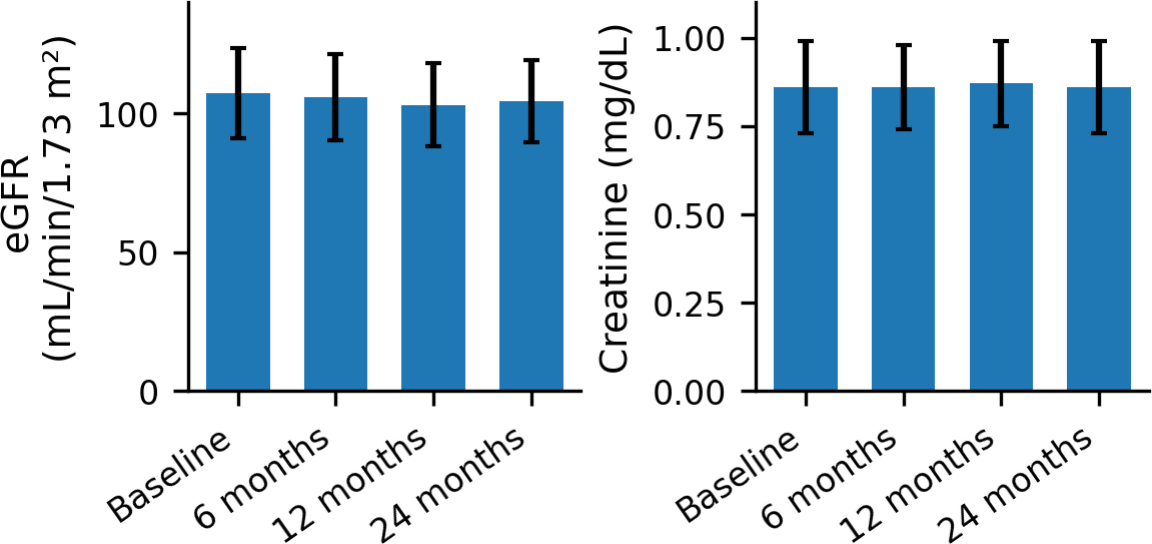
Longitudinal assessment of renal function during digital pre-exposure prophylaxis (PrEP) follow-up in a prospective observational cohort. Temporal evolution of renal function parameters over a 24-month prospective observational follow-up of HIV-negative adults receiving PrEP and monitored through the NAVETA digital telemedicine platform in Spain (n=81). Mean (SD) values of estimated glomerular filtration rate (eGFR; left panel) and serum creatinine (right panel) are shown at baseline, 6, 12, and 24 months. Differences across time points were assessed using Welch ANOVA. AUC: area under the curve; HGB: histogram-based gradient boosting; LR: logistic regression; NPV: negative predictive value; PPV: positive predictive value; RF: random forest.

PrEP was well tolerated, with no participant reporting moderate or severe adverse events. The most frequent patient-reported effects were mild gastrointestinal complaints (68.9%) and mild nervous system symptoms, such as headache, dizziness, tingling, or fatigue (57.8%). All events were transient and self-limited. In the analysis of renal function biomarkers (Figure 1), serum creatinine levels remained stable throughout the follow-up period: 0.86 (SD 0.13) mg/dL at baseline (95% CI 0.83‐0.90), 0.86 (SD 0.12) mg/dL at 6 months (95% CI 0.83‐0.90), 0.87 (SD 0.12) mg/dL at 12 months (95% CI 0.84‐0.93), and 0.86 (SD 0.13) mg/dL at 24 months (95% CI 0.78‐0.93). Data met the assumptions of normality (Shapiro–Wilk, W=0.985, *P*=.055) and homogeneity of variances (Levene test, *P*=.935). The one-way Welch ANOVA showed no statistically significant differences between timepoints (*F*_3, 58.4_=0.252, *P*=.860). In contrast, eGFR values exhibited a slight, nonsignificant decline (*F*_3, 58.4_=0.801, *P*=.498).

Serum creatinine and eGFR (mL/min/1.73 m²) showed a coherent inverse relationship over time. Strong positive correlations were observed between creatinine measurements at consecutive visits (eg, *r*=0.63, *P*<.001 between baseline and 6 mo; *r*=0.84, *P*=.003 between baseline and 24 mo), indicating consistent temporal stability of this biomarker. As expected, creatinine and eGFR values were inversely correlated at all timepoints, with the strongest association at baseline (r=–0.78, *P*<.001). Correlations between creatinine and eGFR at later stages ranged from –0.29 to –0.74. Temporal correlations among eGFR measurements were similarly high (r ≈ 0.73‐0.92, all *P*<.001), reflecting robust internal consistency of renal function indices across the 24-month follow-up (Table 2).

**Table 2.** Pearson correlation matrix between serum creatinine and estimated glomerular filtration rate (eGFR, mL/min/1.73 m²) across follow-up timepoints in a 24-month prospective observational cohort of HIV-negative adults receiving pre-exposure prophylaxis (PrEP) at public hospitals in the Balearic Islands, Spain (n=81). Table values represent Pearson correlation coefficients between serum creatinine and eGFR measured at baseline (BL), 6, 12, and 24 months.

	Creatinine (BL)	Creatinine (6 months)	Creatinine (12 months)	Creatinine (24 months)	eGFR (BL)	eGFR (6 months)	eGFR (12 months)
Creatinine (6 months)	0.628^a^	—^b^	**—**	**—**	**—**	**—**	**—**
Creatinine (12 months)	0.612^a^	0.728^a^	**—**	**—**	**—**	**—**	**—**
Creatinine (24 months)	0.841^a^	0.824^a^	0.560^c^	**—**	**—**	**—**	**—**
eGFR (BL)	−0.779^a^	−0.438^a^	−0.356^c^	−0.531^c^	**—**	**—**	**—**
eGFR (6 months)	−0.392^c^	−0.744^a^	−0.444^c^	−0.571^c^	0.729^a^	**—**	**—**
eGFR (12 months)	−0.358^c^	−0.516^a^	−0.705^a^	−0.457^c^	0.710^a^	0.791^a^	**—**
eGFR (24 months)	−0.671^c^	−0.665^c^	−0.294^c^	−0.775^a^	0.918^a^	0.893^a^	0.752^a^

a *P*<.01.

b Not applicable.

c *P*<.05.

### Analysis of the ePROMs and ePREMs

Patient-reported outcomes (ePROMs and ePREMs) were evaluated longitudinally to characterize changes in psychological well-being, treatment satisfaction, HRQoL, and perceived person-centered care. A detailed summary of mean (SD) values and sample sizes at each timepoint is provided in Table 3.

**Table 3.** Longitudinal trajectories of electronic patient-reported outcome measures (ePROMs) and electronic patient-reported experience measures (ePREMs) over 24 months in a prospective observational cohort of HIV-negative adults receiving pre-exposure prophylaxis (PrEP) and followed through the NAVETA digital monitoring program at public hospitals in the Balearic Islands, Spain (n=81). Numbers (n) indicate the number of participants who completed each questionnaire at each timepoint, reflecting real-world longitudinal follow-up with variable completion rates. *P* values correspond to nonparametric longitudinal comparisons across available timepoints.

ePROMs/ ePREMs and subscale	BL^a^		6 months		12 months		18 months		24 months		*P* value
	mean (SD)	n	mean (SD)	n	mean (SD)	n	mean (SD)	n	mean (SD)	n	
HADS^b^											
Anxiety	5.14 (3.14)	76	3.63 (3.49)	35	4.69 (3.97)	32	4.09 (3.78)	32	4.44 (3.71)	32	.088
Depression	3.16 (2.94)	79	2.34 (2.92)	35	3.03 (3.79)	32	3.44 (3.38)	32	2.44 (2.55)	32	.27
TSQM^c^											
Global satisfaction	—^d^	—	76.2 (17.3)	32	76.2 (8.24)	32	75.7 (15.65)	32	86.6 (12.91)	32	.444
PROMIS-29^e^ (T-score)											
Depression	49 (7.37)	50	—	—	47.7 (7.49)	34	47.6 (7.67)	25	51.4 (9.3)	19	.4
Anxiety	52.1 (7.34)	50	—	—	50.9 (9)	34	50.4 (8.46)	25	52.7 (9.04)	19	.693
Fatigue	44.8 (7.88)	50	—	—	42.9 (10.05)	34	45 (9.8)	25	44.9 (10)	19	.741
Sleep disturbance	47.6 (7.01)	50	—	—	46.4 (7.26)	34	48.2 (7.11)	25	49.5 (5.74)	19	.397
Ability to participate in social roles and activities	54.9 (7.56)	50	—	—	56.2 (8.14)	34	56.7 (7.8)	25	55.3 (7.45)	19	.734
Physical function	56.7 (1.72)	50	—	—	55.8 (3.86)	34	55.7 (4.59)	25	54.5 (5.22)	19	.163
P3CEQ^f^											
Total score	20.1 (6.55)	74	—	—	21 (6.24)	30	21 (7)	23	22.7 (5.28)	19	.39

a BL: baseline.

b HADS: Hospital Anxiety and Depression Scale.

c TSQM: Treatment Satisfaction Questionnaire for Medication.

d Not applicable.

e PROMIS-29: Patient-Reported Outcomes Measurement Information System.

f P3CEQ: Person-Centered Coordinated Care Experience Questionnaire.

Scores on the HADS indicated low levels of psychological distress throughout follow-up. Both anxiety and depression remained within the normal range (0‐8) and showed no statistically significant changes across time (anxiety: *P*=.09; depression: *P*=.27), consistent with emotional stability over the 24-month period.

Treatment satisfaction, assessed through the TSQM Global Satisfaction domain, remained high and stable across follow-up (*P*=.44), reflecting a persistently favorable perception of effectiveness, tolerability, and convenience. The TSQM was not administered at baseline; the first assessment was conducted at 6 months after treatment initiation, as the instrument is specifically designed to evaluate satisfaction with ongoing treatment.

Across PROMIS-29 domains, mean T-scores remained near the population reference mean (50, SD 10). Emotional health dimensions (depression, anxiety, fatigue, sleep disturbance) consistently fell within expected normative limits (<55), whereas social participation and physical function were slightly above average. None of the PROMIS-29 domains showed statistically significant variation over time (all *P*>.05).

Perceived person-centered coordinated care, measured using the P3CEQ total score, showed a modest but nonsignificant upward trend over follow-up (*P*=.39), indicating stable or slightly improved perceptions of coordination, communication, and shared decision-making. In addition, satisfaction with the NAVETA telemedicine follow-up model, assessed using a platform-specific Likert-type satisfaction scale, remained consistently high throughout follow-up (mean score 8/10), reflecting sustained positive user evaluations of the digital monitoring experience.

To assess potential bias related to missing data, individual completion patterns across scheduled assessment stages (baseline, 6, 12, 18, and 24 mo) were examined. Questionnaire completion decreased progressively over follow-up: 94.8% of participants completed at least 25% of assessments, 76.3% completed ≥50%, 51.5% completed ≥75%, and 30.9% completed all scheduled stages. Individual completion trajectories and summary completion ratios are presented in Figure S1 and Table S2 in Multimedia Appendix 1. To evaluate whether early noncompletion was associated with underlying clinical or psychological differences, baseline ePROMs and ePREMs scores were compared between participants with low completion (≤25% of stages; n=5) and high completion (≥75%; n=50). No statistically significant differences were observed across any domain (HADS, TSQM, PROMIS-29, or P3CEQ; all >.25), and baseline score distributions were closely aligned between groups.

### Correlations Among ePROMs/ePREMs During Follow-Up

Correlation analyses among patient-reported outcomes (PROMs and PREMs) revealed consistent interdomain relationships over time. At baseline, moderate associations were observed between overall HRQoL (PROMIS-29) and both psychological well-being (HADS, *r*=0.58) and treatment satisfaction (TSQM, *r*=–0.61), indicating that better emotional status was linked to higher perceived quality of life and satisfaction. Person-centered coordinated care (P3CEQ) showed weak positive correlations with other measures. At 24 months, the pattern strengthened, with high correlations between HADS and PROMIS-29 (*r*≈1.0) and inverse associations with TSQM (*r*≈–0.72), suggesting that improved emotional well-being paralleled higher HRQoL and treatment satisfaction. These findings highlight internal consistency across PROM domains and the mutual influence of emotional, experiential, and satisfaction-related dimensions during follow-up.

### Analysis of the Response Profile to Patient-Reported Outcomes Questionnaires

**Figure 2.**
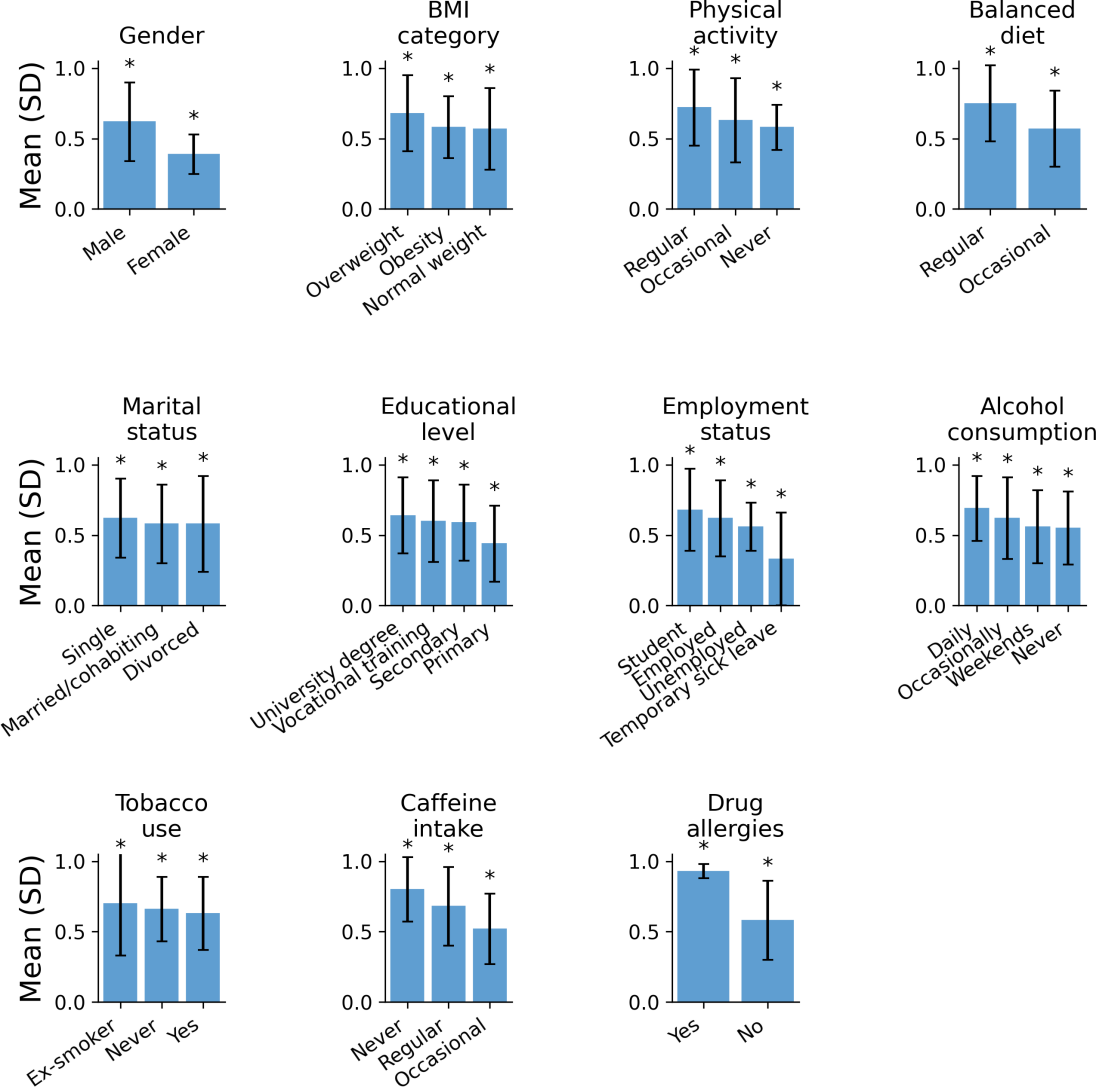
Sociodemographic and behavioral determinants of digital engagement during a 24-month telemedicine-based PrEP follow-up in a prospective observational cohort. *P<.05.

Engagement to the Naveta digital follow-up platform exhibited significant sociodemographic and behavioral gradients (Figure 2). Men demonstrated substantially higher engagement than women (0.62, SD 0.28 vs 0.39, SD 0.14; *P*<.001), and engagement increased progressively with age, being significantly greater in the 46‐65 and 66‐75 years age groups compared with younger participants (both *P*<.001, Dunn test). Participants classified as overweight also showed higher engagement than those with normal weight (*P*<.001), although no differences were found between overweight and obese categories.

Lifestyle-related variables were among the strongest determinants of engagement. Those reporting regular physical activity (0.72, SD 0.27) exhibited significantly higher engagement compared with participants who were inactive (*P*<.001) or exercised occasionally (*P*=.001). Similarly, maintaining a balanced diet was associated with greater continuity than occasional engagement to healthy eating habits (*P*<.001). Educational level displayed a graded pattern: participants with university degrees showed higher engagement than those with primary or secondary education (*P*=.005 and *P*=.03, respectively). Employment status also played a major role, as individuals on temporary sick leave (0.33, SD 0.33) reported markedly lower engagement than employed (*P*<.001) or student participants (*P*<.001).

Behavioral factors related to substance use followed similar trends. Daily alcohol consumers presented higher engagement than those drinking only on weekends or never (*P*=.003). Former smokers were more adherent than current smokers (*P*=.007), and complete caffeine abstainers showed the highest engagement levels (0.80, SD 0.23), significantly exceeding both regular (*P*<.001) and occasional consumers (*P*<.001).

Sociodemographic and behavioral gradients of digital engagement among HIV-negative adults receiving PrEP and followed through the NAVETA digital telemedicine platform at public hospitals in the Balearic Islands, Spain (n=81). Bars represent mean (SD) engagement ratios, defined as the proportion of completed ePROMs/ePREMs over scheduled assessments. Asterisks indicate statistically significant between-group differences based on nonparametric comparisons (Kruskal–Wallis test with Dunn post hoc correction; *P*<.05).

**Figure 3.**
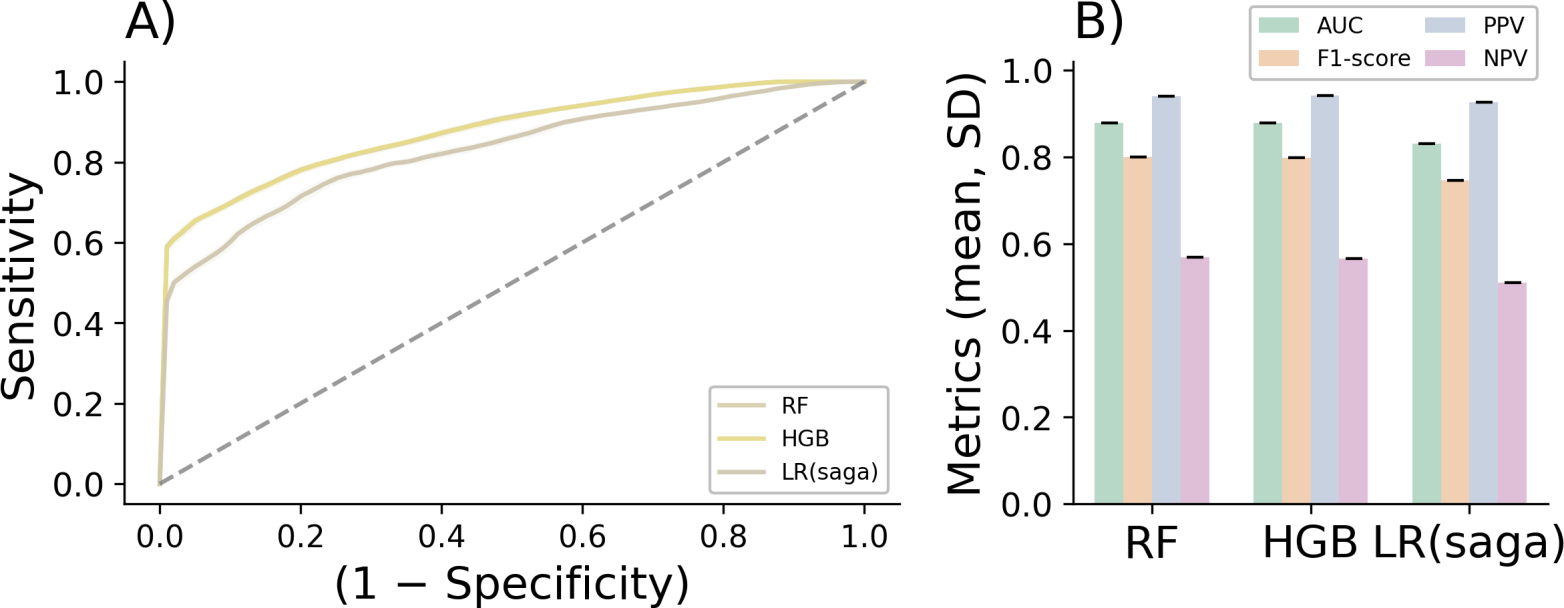
Discrimination performance of machine learning models for predicting digital engagement during pre-exposure prophylaxis (PrEP) follow-up in a prospective observational cohort. Comparative performance of machine learning models for questionnaire-level digital engagement classification in a prospective observational cohort of HIV-negative adults receiving PrEP and followed through the NAVETA digital telemedicine platform at public hospitals in the Balearic Islands, Spain (n=81; 24-mo follow-up). (**A**) Receiver operating characteristic (ROC) curves showing the mean performance of HGB, RF, and LR (saga solver) across repeated stratified 5×5 cross-validation. The y-axis represents sensitivity (true positive rate) and the x-axis represents 1 − specificity (false positive rate). (**B**) Summary of classification metrics (mean, SD) across cross-validation folds, including area under the curve (AUC), *F*_1_-score, PPV, and NPV. AUC: area under the curve; HGB: histogram-based gradient boosting; LR: logistic regression; NPV: negative predictive value; PPV: positive predictive value; RF: random forest.

Although the bivariate associations were statistically robust, classical regression models were insufficient to capture the underlying nonlinear patterns governing digital engagement [34]. For this reason, we applied our machine learning framework, ALGOPROMIA-Classification, to improve discrimination between engagement profiles within the NAVETA follow-up program (Figure 3, Table 4).

**Table 4.** Performance metrics of machine learning models for predicting digital engagement during a 24-month follow-up in a prospective observational cohort of HIV-negative adults receiving pre-exposure prophylaxis (PrEP) and monitored through the NAVETA digital telemedicine platform at public hospitals in the Balearic Islands, Spain (12,299 questionnaire-level events). Performance metrics correspond to questionnaire-level digital engagement classification. Engagement was defined as a binary outcome (engaged vs nonengaged). All metrics are reported as mean (SD) across repeated stratified 5×5 cross-validation. Class imbalance was preserved across training and test folds.

Model	AUC^a^, mean (SD)	Accuracy, mean (SD)	Precision, mean (SD)	Recall, mean (SD)	*F*_1_-score, mean (SD)	PPV^b^, mean (SD)	NPV^c^, mean (SD)
RF^d^	0.817 (0.007)	0.704 (0.007)	0.919 (0.007)	0.637 (0.008)	0.753 (0.006)	0.919 (0.007)	0.500 (0.007)
HGB^e^	0.816 (0.007)	0.704 (0.007)	0.919 (0.007)	0.637 (0.008)	0.752 (0.006)	0.919 (0.007)	0.499 (0.006)
LR^f^ (saga)	0.809 (0.007)	0.705 (0.007)	0.918 (0.007)	0.638 (0.008)	0.753 (0.006)	0.918 (0.007)	0.499 (0.007)

a AUC: area under the receiver operating characteristic curve.

b PPV: positive predictive value.

c NPV: negative predictive value.

d RF: random forest.

e HGB: histogram-based gradient boosting.

f LR: logistic regression.

Although the cohort consisted of 81 individuals, the machine learning models were trained on all available questionnaire–response events (12,299 observations), as engagement was defined at the event level rather than the participant level (Table S1 in Multimedia Appendix 1).

Across repeated stratified 5×5 cross-validation, ensemble-based algorithms again showed the strongest overall predictive performance (Figure 1, Table 4). RF and HGB achieved very similar levels of discrimination (mean AUC=0.817, SD 0.007 and 0.816, SD 0.007, respectively), with comparable accuracy (≈0.70) and *F*_1_-scores (≈0.75). Both ensemble models exhibited high positive predictive values (≈0.92), reflecting a strong ability to correctly identify engaged participants, whereas negative predictive values (NPVs) were consistently more modest (NPV≈0.50). Logistic regression (saga) showed slightly lower discrimination (AUC=0.809, SD 0.007), while achieving similar accuracy and *F*_1_ performance. Taken together, these results suggest that ensemble-based approaches offered a small but consistent advantage in modeling the nonlinear behavioral patterns associated with sustained engagement in digital PrEP monitoring.

**Figure 4.**
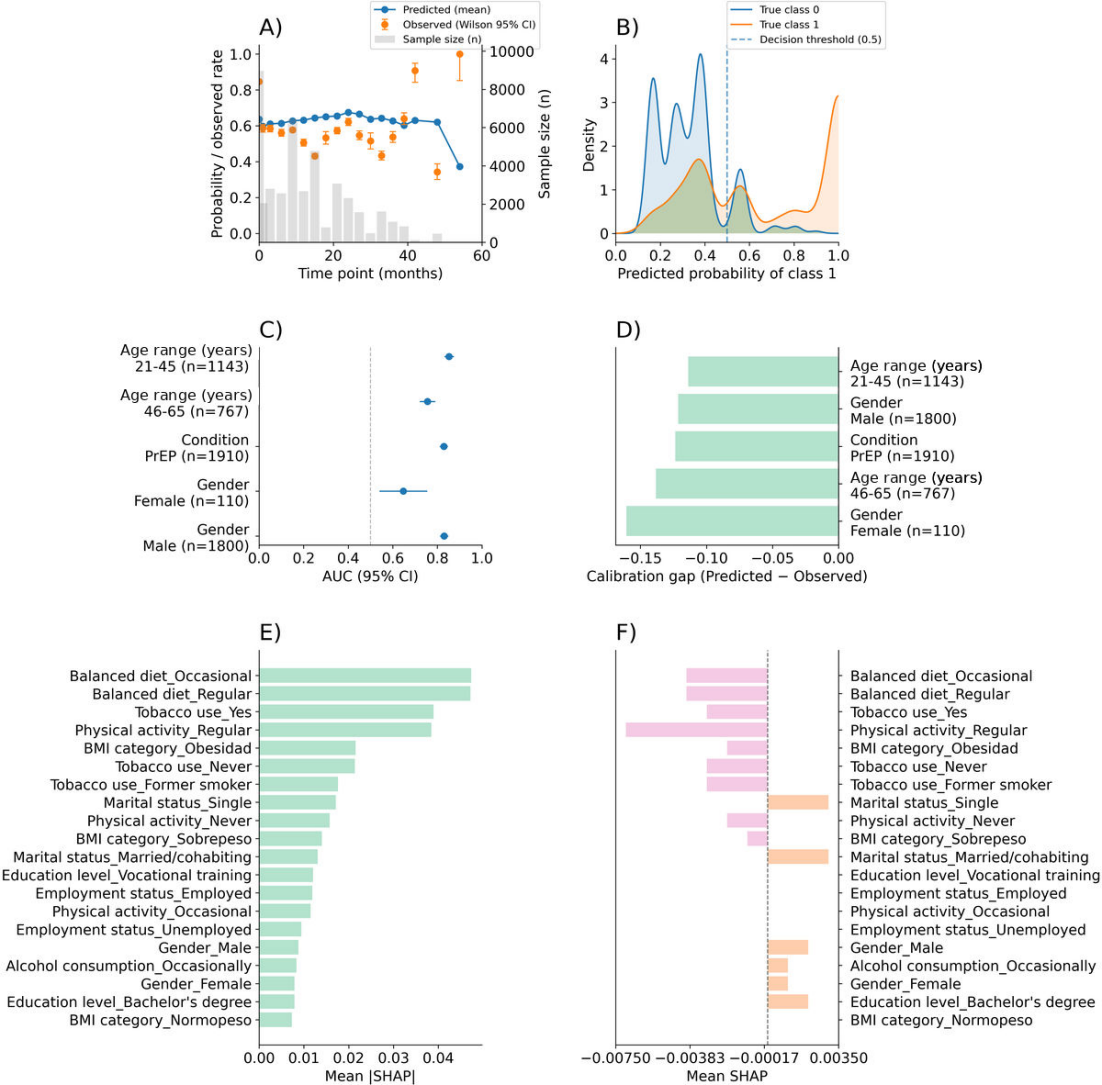
Temporal stability, subgroup performance, and explainability of the random forest model for predicting digital engagement during a 24-month PrEP follow-up. Evaluation of a random forest classifier applied to questionnaire-level data from a prospective observational cohort of HIV-negative adults receiving PrEP and monitored through the NAVETA digital telemedicine platform at public hospitals in the Balearic Islands, Spain (81 participants contributing 12,299 questionnaire-level observations). (**A**) Temporal stability of predicted engagement probabilities compared with observed engagement rates across follow-up timepoints, with 95% Wilson CIs; gray bars indicate the number of observations per timepoint. (**B**) Distribution of predicted engagement probabilities for engaged and nonengaged observations shown using kernel density estimates; the dashed vertical line indicates the fixed decision threshold (0.5). (**C**) Discriminatory performance across predefined demographic subgroups (sex and age), expressed as AUC with 95% CIs. (**D**) Subgroup-level calibration, expressed as the difference between predicted and observed engagement probabilities (calibration gap). (**E**) Global feature importance derived from permutation-based SHAP analysis, ranking predictors by mean absolute SHAP values. (**F**) Directional contribution of predictors to model output, expressed as mean signed SHAP values. All analyses were conducted at the questionnaire level, with individual participants contributing multiple observations across follow-up. AUC: area under the curve; PrEP: pre-exposure prophylaxis; SHAP: Shapley Additive Explanations.

On the basis of this comparative evaluation, the RF model was retained for downstream analyses. Model performance and behavior were then characterized in greater detail using the full set of questionnaire-level observations, including temporal stability of predicted versus observed engagement, score separation, subgroup-level discrimination and calibration, and permutation-based SHAP explainability (Figure 4). All analyses were conducted at the questionnaire level; accordingly, n refers to the number of questionnaire-level observations (digital interaction events), with individual participants contributing multiple observations across follow-up stages when applicable.

Across monitoring stages (Figure 4A), the mean predicted probability of engagement remained relatively stable over time, ranging from 0.607 to 0.669 across the most frequently observed timepoints. Across all time points, the mean difference between observed and predicted engagement rates was −0.022, indicating a small average deviation. Observed engagement rates varied more widely (0.430‐0.846). At baseline (0 mo; n=1910), the observed engagement rate was 0.846, and the model-predicted mean probability was 0.636. At 6 months (n=1817), predicted and observed values were closely aligned (0.617 vs 0.616). At 12 months (n=1583), the predicted probability was 0.610, and the observed rate was 0.648. At 15 months (n=1693), the predicted probability was 0.607 while the observed rate was 0.430. At 24 months (n=1235), predicted and observed values again showed close agreement (0.616 vs 0.609). The sample size decreased over the follow-up stages.

Predicted score distributions showed partial separation between engaged and nonengaged observations (Figure 4B). The AUC derived from the predicted probabilities was 0.816. Among engaged observations (class 1; n=1288), the median predicted probability was 0.711 (IQR 0.464‐1.000); among nonengaged observations (class 0; n=622), the median was 0.321 (IQR 0.174‐0.607). Using a fixed decision threshold of 0.5, sensitivity was 0.637 and specificity was 0.851 (false positive rate 0.149).

Subgroup analyses indicated comparable discrimination across sex and age strata (Figure 4C). The AUC was 0.825 in men (n=1800; 95% CI 0.800‐0.850) and 0.795 in women (n=110; 95% CI 0.721‐0.870). By age group, the AUC was 0.783 in participants aged 21‐45 years (n=1143; 95% CI 0.755‐0.811) and 0.835 in those aged 46‐65 years (n=767; 95% CI 0.807‐0.863). Calibration gaps (predicted minus observed engagement probability) were −0.037 in men, −0.161 in women, −0.090 in the 21‐45 year group, and −0.018 in the 46‐65 year group (Figure 4D).

Permutation-based SHAP analysis identified lifestyle-related variables as the most influential predictors of engagement (Figure 4E). The largest mean absolute SHAP values corresponded to a balanced diet (occasional and regular; mean |SHAP|=0.047 for both), tobacco use (yes: 0.039; never: 0.021; former smoker: 0.018), and regular physical activity (mean |SHAP|=0.039). BMI category (obesity) showed mean |SHAP|=0.021. Mean signed SHAP values indicated variable directionality across predictors (Figure 4F). Regular physical activity had a mean signed SHAP of −0.0075 with a proportion of positive contributions of 0.484. A balanced diet showed mean signed SHAP values of −0.0037 (occasional) and −0.0038 (regular), with positive contribution proportions of 0.343. Marital status (single) showed a mean signed SHAP of +0.00345 with a positive contribution proportion of 0.726, despite a lower overall importance (mean |SHAP|=0.0173).

## Discussion

### Principal Findings

This study provides descriptive evidence that long-term digital follow-up of PrEP users through integrated ePROMs and ePREMs is operationally feasible and that participants who remained engaged in the program reported stable psychological well-being, experiential outcomes, and HRQoL over 24 months. Across follow-up, no clinically relevant fluctuations were observed in anxiety, depression, treatment satisfaction, or HRQoL domains, suggesting that the cohort, as observed, did not experience deterioration in emotional or quality-of-life outcomes. In parallel, the application of a machine learning–based framework enabled detailed characterization of digital engagement patterns at the questionnaire level, identifying sociodemographic, behavioral, and lifestyle-related factors associated with sustained participation.

Together, these findings address the study objectives by demonstrating the feasibility of digitally supported PrEP monitoring using ePROMs/ePREMs, and by illustrating how predictive analytics can complement descriptive clinical data to better understand engagement dynamics in real-world preventive care.

### Clinical Outcomes, HRQoL, and Patient-Reported Experiences

When HRQoL outcomes were examined, participants exhibited stable psychological and HRQoL profiles throughout the monitoring period. The lack of clinically relevant fluctuations across anxiety, depression, satisfaction, and HRQoL domains indicates that the cohort, as observed, did not experience deterioration in emotional well-being over time. These findings are consistent with prior reports suggesting psychological stability among PrEP users [1,2], although comparisons should be interpreted cautiously given differences in study design.

Across PROMIS-29 domains, mean T-scores remained close to the normative population range, indicating preserved HRQoL throughout follow-up. Emotional domains (depression, anxiety, fatigue, and sleep disturbance) showed values within expected ranges, in line with the consistently low and stable HADS scores observed across all visits (normal range 0‐7). These descriptive patterns are consistent with literature characterizing PrEP users as generally psychosocially stable populations engaged in care [3].

Treatment satisfaction, assessed using the TSQM, remained stable throughout follow-up, in line with earlier observational findings reporting high satisfaction with effectiveness, convenience, and tolerability among PrEP users [4,5]. In addition, perceived person-centered coordinated care, measured with the P3CEQ, showed a modest, nonsignificant upward trend, potentially reflecting increasing familiarity with the digital platform or continuity of interactions with health care professionals, although no inference can be made regarding the effect of digital care on these perceptions.

Correlation analyses among patient-reported outcomes revealed coherent interdomain associations at baseline and at 24 months. HADS and PROMIS-29 emotional domains showed moderate relationships (eg, *r*=0.58), and treatment satisfaction demonstrated inverse associations with psychological distress (*r*=–0.61). By 24 months, these associations were slightly stronger, although the observational design precludes interpretation regarding temporal causality or directional effects.

### Clinical and Organizational Value of Digital Monitoring

Importantly, the clinical value of the digital monitoring framework in this study does not lie in detecting large group-level changes—which were not observed—but rather in enabling individual-level risk stratification and care reconfiguration that would not have been feasible under standard visit-based follow-up. The NAVETA platform incorporates a configurable clinical dashboard, conceptually analogous to established systems such as AmbuFlex, including automated alerts for ePROMs scores deviating from predefined normative ranges [35,49]. This functionality allows clinicians to rapidly identify individuals requiring closer attention while maintaining remote oversight of stable patients.

In practice, this digital visibility supported a shift from a rigid, hospital-centric follow-up model toward a responsive, risk-based care pathway. For individuals showing stable ePROMs profiles across physical and psychological domains, clinicians were able to safely reduce routine in-person visits and transition follow-up to a predominantly digital format. This, in turn, enabled decentralized medication dispensing, allowing stable PrEP users to collect treatment at their nearest primary care center rather than attending hospital pharmacy services. Conversely, when moderate or clinically relevant deviations in ePROMs domains were detected at the individual level, the system facilitated expedited telephone or in-person assessments, ensuring timely clinical review despite the absence of statistically significant changes at the group level.

Although this study was not designed to formally quantify the frequency or outcomes of such individual-level interventions, the findings illustrate how clinically meaningful signals can emerge at the patient level even when aggregate trajectories remain stable, highlighting a limitation of relying exclusively on group-level statistical significance to judge clinical utility.

From an implementation perspective, the digital follow-up model was designed to minimize operational burden. Questionnaire administration and scoring were automated, with staff effort concentrated primarily on patient onboarding and periodic review of flagged cases. While no formal cost-effectiveness analysis was conducted, the platform enabled avoidance of unnecessary hospital visits, reduction in patient travel and work absenteeism, and more efficient allocation of clinician time toward individuals with complex needs. These practical efficiencies are supported by consistently high satisfaction ratings regarding the fully telemedicine-based follow-up model, as captured through NAVETA-specific ePROMs and ePREMs.

Taken together, the present findings suggest that the primary added value of intensive digital monitoring in PrEP care lies not in altering average clinical outcomes over time, but in optimizing care delivery, resource allocation, and patient convenience while maintaining clinical safety.

### Digital Engagement Patterns and Machine Learning Analysis

While these descriptive analyses characterize overall clinical stability, understanding patterns of digital engagement requires analytical approaches capable of capturing multivariable and nonlinear relationships across repeated observations.

Nonparametric comparisons of engagement across sociodemographic and behavioral variables revealed marked gradients in participation patterns. Men, older participants, individuals reporting healthier lifestyle habits, and those with higher educational attainment or active employment status tended to exhibit greater continuity in digital engagement. These patterns are consistent with previous studies highlighting structural, behavioral, and socioeconomic determinants of participation in digital health interventions [7,8]. However, given the observational design, these associations should be interpreted strictly as correlational and not as evidence of causal effects.

To further characterize the multivariable relationships underlying engagement, we applied the ALGOPROMIA-Classification machine learning framework. Ensemble-based models, including HGB and RF, demonstrated good discriminatory performance, with AUC values around 0.82, indicating a consistent ability to distinguish between engaged and less engaged questionnaire-level observations. Notably, all evaluated algorithms showed very similar performance across discrimination and classification metrics. This convergence likely reflects the structure of the data, characterized by class imbalance and a relatively limited number of dominant predictors [50]. Under these conditions, both linear and nonlinear models can capture the main signal once appropriate regularization, oversampling within training folds, and repeated cross-validation are applied. Consequently, model performance appears to be driven more by the underlying data patterns than by algorithmic complexity per se. The small numerical differences observed between models should therefore be interpreted cautiously, as they primarily reflect averaging across repeated cross-validation folds and rounding precision rather than substantively meaningful differences in predictive capability [51].

On the basis of the comparative model evaluation, the RF algorithm was selected for downstream analyses. This choice was not driven by superior point estimates of discrimination or accuracy, as all evaluated algorithms showed highly comparable performance across metrics. Instead, RF was retained to ensure methodological consistency with previous applications of the ALGOPROMIA framework in related clinical contexts, and because it offers a favorable balance between robustness, interpretability, and stability under class imbalance and repeated cross-validation. Given the convergence in performance across models, this selection reflects a pragmatic and reproducible modeling strategy rather than an optimization-driven decision [52,53].

Model behavior was subsequently examined in greater depth using the full set of questionnaire-level observations, focusing on temporal stability of predicted versus observed engagement, score separation, subgroup-level discrimination and calibration, and permutation-based SHAP explainability (Figure 4). Across monitoring stages, the mean predicted probability of engagement remained relatively stable over time. The average difference between observed and predicted engagement rates across all stages was −0.022, indicating a small overall deviation between model estimates and empirical data. In contrast, observed engagement rates exhibited greater variability (range 0.430‐0.846). While prediction and observation were closely aligned at several time points (notably at 6 and 24 mo), larger discrepancies were observed at baseline and at 15 months. Despite the overall temporal stability observed, engagement dynamics at specific monitoring stages remain to be fully characterized.

Analysis of predicted score distributions demonstrated partial separation between engaged and nonengaged observations, with an AUC of 0.816 indicating good overall discrimination. However, the operating characteristics of the model revealed an asymmetric performance profile. When applying a fixed decision threshold of 0.5, the classifier prioritized specificity over sensitivity, resulting in high specificity (0.851) and positive predictive value, but more modest sensitivity (0.637) and NPV. From a clinical and implementation perspective, this performance profile is not necessarily optimal for digital follow-up programs, where the primary objective is often the early identification of individuals at risk of disengagement. In this context, false negatives—participants predicted as engaged who subsequently fail to respond—are more consequential than false positives, as they represent missed opportunities for timely intervention.

This asymmetric performance likely reflects both the underlying class imbalance and the limited availability of informative longitudinal behavioral signals early in follow-up. Because engagement is a dynamic and evolving process, richer temporal information—capturing changes in interaction patterns, response latency, and behavioral trajectories over time—may be required to improve sensitivity for disengagement prediction. Accordingly, these findings support the need for iterative model updating as additional longitudinal data become available.

Subgroup analyses showed broadly comparable discriminatory performance across sex and age strata. Although AUC values differed numerically between groups, the CIs overlapped substantially, providing no evidence of systematic performance degradation in any specific subgroup. Calibration gaps were generally small and negative, indicating a tendency toward slight underestimation of engagement probability, particularly among women and younger participants. However, the limited number of women reduces statistical precision in sex-stratified analyses and constrains the generalizability of subgroup-specific estimates, warranting confirmation in more sex-balanced PrEP cohorts [35,54-56].

### Explainability and Interpretation of SHAP Results

Permutation-based SHAP analysis provided further insight into the drivers of model predictions. Consistent with prior research in telemedicine and digital health engagement, lifestyle-related variables emerged as the most influential contributors to sustained participation [57,58]. In the present model, indicators of balanced diet, tobacco use, and physical activity exhibited the largest mean absolute SHAP values, highlighting their relative importance for engagement prediction at the questionnaire level. However, mean absolute SHAP values reflect relative importance within the model rather than clinically meaningful changes in engagement probability. SHAP values quantify feature contributions on the model’s internal scale and are intended for comparative interpretation across variables, not for direct translation into probability differences or effect sizes.

Importantly, mean signed SHAP values were frequently close to zero, indicating substantial heterogeneity in the direction of feature effects across individuals [59]. For example, regular physical activity showed high global importance but mixed directional influence, contributing positively in some observations and negatively in others. In contrast, certain predictors with lower overall importance—such as marital status (single)—displayed more consistent directional effects.

Taken together, these patterns align with existing evidence linking health-related behaviors to adherence and continuity in digital follow-up programs, while underscoring that such associations should be interpreted strictly as model-based and noncausal. The relatively small magnitude of individual SHAP contributions does not imply that engagement behavior is stochastic or unpredictable. Rather, it reflects the multifactorial and context-dependent nature of digital engagement, particularly when modeled at the questionnaire level [60]. Engagement decisions are shaped by a combination of stable individual characteristics and dynamic situational determinants not explicitly captured in the present model.

Importantly, this work is subject to several limitations inherent to its observational, single-arm design. In the absence of a control group or structured pre–post comparisons, no causal inferences can be drawn regarding the effects of PrEP use or the NAVETA digital monitoring system on psychological, experiential, or HRQoL outcomes. The stable trajectories observed may reflect baseline characteristics of individuals who initiated PrEP or who remained engaged in follow-up, rather than effects attributable to digital monitoring itself. Selection bias and a potential healthy-user effect must therefore be considered, particularly in light of the 19.8% discontinuation rate observed during follow-up.

A key methodological consideration relates to missing data and its potential influence on longitudinal interpretations. Completion rates decreased over the scheduled assessment stages, as expected in a real-world telemedicine context [61]. Although exploratory analyses did not indicate systematic differences in baseline HRQoL or psychological status between participants with high and low questionnaire completion, residual bias cannot be fully excluded. The voluntary nature of ePROMs/ePREMs completion implies that observed longitudinal patterns may over-represent individuals who are more digitally engaged or motivated, limiting generalizability.

Finally, engagement modeling was performed at the questionnaire level and did not incorporate richer temporal or contextual variables that may influence response behavior, such as transient life events or short-term changes in perceived relevance of questionnaires.

### Conclusions

Taken together, the present findings indicate that digitally supported PrEP follow-up using integrated ePROMs and ePREMs is feasible in routine clinical practice and can support stable long-term monitoring of psychological well-being, HRQoL, and treatment satisfaction. Beyond descriptive clinical stability, the combination of patient-reported data and explainable machine learning provides actionable insight into engagement patterns, enabling more adaptive, resource-efficient, and person-centered models of preventive care.

While causal effects cannot be inferred, this study demonstrates how digital monitoring infrastructures can be leveraged to optimize care delivery, prioritize clinical attention where needed, and generate high-resolution engagement data to inform future intervention strategies. These results establish a foundation for subsequent comparative, interventional, and cost-effectiveness studies aimed at evaluating the broader impact of digital monitoring on PrEP care pathways.

## Supplementary material

10.2196/87592 Multimedia Appendix 1 Participant-level completion trajectories and observation counts across scheduled ePROM and ePREM assessment stages during 24-month digital follow-up.

## Abbreviations

AUC: area under the curve
eGFR: estimated glomerular filtration rate
ePREM: electronic patient-reported experience measure
ePROM: electronic patient-reported outcome measure
HADS: Hospital Anxiety and Depression Scale
HGB: histogram-based gradient boosting
HRQoL: health-related quality of life
NPV: negative predictive value
P3CEQ: Person-Centered Coordinated Care Experience Questionnaire
PrEP: pre-exposure prophylaxis
PROMIS-29: Patient-Reported Outcome Measurement Information System Profile-29
RF: random forest
SHAP: Shapley Additive Explanations
TSQM: Treatment Satisfaction Questionnaire for Medication
