# Supplementary material for: Digital Monitoring of Pre-Exposure Prophylaxis Users Through Electronic Patient-Reported Outcome Measures and Electronic Patient-Reported Experience Measures: Multicenter Prospective Study on Feasibility, Safety, and Predictive Modeling of Digital Engagement
Source: J Med Internet Res. 2026 Apr 13;28:e87592. doi: 10.2196/87592 (PMC13075468; doi:10.2196/87592)
Supplement: Multimedia Appendix 1 [file jmir-v28-e87592-s001.docx]

**Diagrams/ Figures for multimedia sections:**

**Table S1: The study survey**

| **Section 1: Respondent characteristics**  The first six questions help assess if this survey's respondents are as diverse as the European population.  The answers may also help work out how to best address the needs and preferences of subgroups. | | |
| --- | --- | --- |
|  | **Question** | **Respondents choice** |
| 1 | In which country do you live (primarily)? | Austria, Belgium, Bulgaria, Croatia, Cyprus, Czechia, Denmark, Estonia, Finland, France, Germany, Greece, Hungary, Iceland, Ireland, Italy, Latvia, Liechtenstein, Lithuania, Luxembourg, Malta, Netherlands, Norway, Poland, Portugal, Romania, Slovakia, Slovenia, Spain, Sweden, Switzerland, United Kingdom, Ukraine, Other |
| 2 | What year were you born? (Participants of this study should be 18 years or older) | Drop down of years up to 2005 |
| 3 | What is your gender? | - Male - Female - Other |
| 4 | What is your education level? | - Elementary school or less - High school - Practical/ vocational education - Bachelor/ Master/ Doctor or similar |
| 5 | In general, would you say your health is… | - Poor - Fair - Good - Very good |
| 6 | Do you provide informal care? (Informal care is care for a chronically ill, disabled, or elderly family member or friend) | - Yes, I provide informal care every day - Yes, I provide informal care at least once a week - Yes, I provide informal care, but not every week - No, I do not provide informal care |
| **Section 2: Use**  Combined with the first questions this next question tells who uses which type of health apps (already). | | |
| 7 | Which type of health apps do you use or have you used in the past? (More than one answer possible) | - COVID-19 app - Health insurance app - Hospital or clinic app (apps to schedule a visit, to see your medical record, etc.) - Personal health record app (apps to document your health issues, allergies, medication, blood type, organ donor preferences, contact in case of emergency, etc.) - Activity app (apps to track your number of steps, workouts, running, etc.) - Nutrition app (apps to track what you eat, your body weight, etc.) - Sleep app (apps to track your sleep) - Menstruation app (apps to track and predict your period, your most fertile days, etc.) - Mindfulness app - Vital signs app (apps to track your blood pressure, heart rate, body temperature, breathing rate) - Disease management apps (apps to learn more about your disease, apps to help take your medication in time, apps to track your symptoms, etc., for example, diabetes apps, cancer apps, heart disease apps) - Informal caregiver app (apps to help a chronically ill, disabled, or elderly family member or friend with their health and care) - Research apps (apps to participate in medical research) - Diagnostic app (apps to assist in diagnosing a medical condition, for example, checking your skin to detect cancer, measuring your heart rate to detect a heart condition, etc.) - Treatment apps (physiotherapy apps, rehabilitation apps, apps to treat mental conditions such as depression, etc.) - Other, Please mention which other health apps you use. - I do not use a health app |
| **Section 3: Trust**  The next two questions explore whose recommendations you use and trust in choosing health apps.  Your answers indicate who can help distribute information materials about choosing health apps. | | |
| 8 | Whose advice/tips have you used in the past to choose a health app? (more than one answer possible) | - A health professional (medical doctor, nurse, physiotherapist, etc.) - A pharmacist - A health app library - A government or health authority (website, brochure, commercial, etc.) - A health professional organization (website, brochure, commercial, etc.) - A patient organization (website, brochure, commercial, etc.) - A peer support group (for example Facebook group for patients with a specific health issue) - My family/friends - Traditional media (newspapers, magazines, radio, television) - Personal social media posts (Facebook, Instagram, etc., including influencers) - The App Store or Google Play store (Includes manufacturer information, user reviews, and order the apps are displayed) - A Google search - A health app manufacturer (includes advertisements) - Other, Please mention whose other advice/tips you used - None of the above |
| 9 | Would you trust advice/tips for a health app from.......   - A health professional (medical doctor, nurse, physiotherapist, etc.)? - A pharmacist ? - A health app library - A government or health authority (website, brochure, commercial, etc.) - A health professional organization (website, brochure, commercial, etc.) - A patient organization (website, brochure, commercial, etc.) - A peer support group (for example Facebook group for patients with a specific health issue) - My family/friends - Traditional media (newspapers, magazines, radio, television) - Personal social media posts (Facebook, Instagram, etc., including influencers) - The App Store or Google Play store (Includes manufacturer information, user reviews, and order the apps are displayed) - A Google search - A health app manufacturer (includes advertisements) - Other, Please mention whose other advice/tips you use - None of the above | - I do not trust - I am not sure if I can or should trust - I trust - I do not know or have not thought about it |
| **Section 4: Review and rate**  Some European governments have adopted a method to review and rate health apps, others have not.  Your answer helps Label2Enable address thoughts on reviewing and rating health apps with governments.  If you have anything further to add then please detail your thoughts and comments in the last question. | | |
| 10 | Do you think the government should review and rate health app quality to help you choose a health app?  (Government includes health authorities such as the Ministry of Health.  Health app quality includes if the app benefits health, is safe, easy to use, reliable, secures data, etc.) | - Yes, I think the government should review and rate health app quality - No, but I think the government should pay another organization to review and rate health app quality - No, I think the government should not review and rate health app quality and should not pay another organization to review and rate health app quality |
| 11 | Any final thoughts or comments you would like to add? |  |
| Thank you for your participation.  You can find the results of this survey in early 2023 on the Label2Enable website ([www.label2enable.eu](http://www.label2enable.eu)). | | |

**Table S2:** Countries of respondents

| **Country** | **n (%)** |
| --- | --- |
|  |  |
| The Netherlands | 403 (32.8) |
| Germany | 94 (7.7) |
| Spain | 89 (7.2) |
| Belgium | 84 (6.8) |
| Malta | 73 (5.9) |
| Croatia | 68 (5.5) |
| Lithuania | 56 (4.6) |
| Bulgaria | 45 (3.7) |
| Italy | 33 (2.7) |
| Greece | 32 (2.6) |
| Slovakia | 24 (2.0) |
| France | 21 (1.7) |
| United Kingdom | 19 (1.5) |
| Sweden | 18 (1.5) |
| Austria | 17 (1.4) |
| Norway | 16 (1.3) |
| Denmark | 15 (1.2) |
| Ireland | 15 (1.2) |
| Latvia | 15 (1.2) |
| Portugal | 15 (1.2) |
| Romania | 14 (1.1) |
| Switzerland | 11 (0.9) |
| Poland | 10 (0.8) |
| Estonia | 8 (0.7) |
| Other | 8 (0.7) |
| Slovenia | 6 (0.5) |
| Ukraine | 6 (0.5) |
| Luxembourg | 4 (0.3) |
| Cyprus | 3 (0.2) |
| Czechia | 2 (0.2) |
| Finland | 2 (0.2) |
| Hungary | 1(0.1) |
| Iceland | 1 (0.1) |
| Liechtenstein | 0 (0.0) |
| Total | 1228 (100) |

**Multimedia Appendix 3:**

Figure S1. Whose advice/tips do participants trust to choose a health app?
